# Editorial: Advancements in intraoperative optical technologies for neurosurgery guidance

**DOI:** 10.3389/fnins.2024.1527174

**Published:** 2024-12-04

**Authors:** Bruno Montcel, Charly Caredda, Pablo A. Valdés

**Affiliations:** ^1^Univ Lyon, INSA-Lyon, Université Claude Bernard Lyon 1, UJM-Saint Etienne, CNRS, Inserm, CREATIS UMR 5220, U1294, Lyon, France; ^2^Department of Neurosurgery, University of Texas Medical Branch, Galveston, TX, United States; ^3^Department of Neurobiology, University of Texas Medical Branch, Galveston, TX, United States; ^4^Department of Electrical and Computer Engineering, Rice University, Houston, TX, United States

**Keywords:** intraoperative optical technologies, hyperspectral optical imaging, fluorescence spectroscopy, polarization imaging, neurosurgical guidance

Intraoperative optical technologies have shown immense promise as neurosurgical adjuncts. These technologies provide real-time feedback, are cost-effective, and seamlessly integrate into the surgical workflow, making them valuable tools for enhancing surgical guidance and tissue assessment. Various novel optical imaging modalities can be seen have been developed such as color, multispectral, and hyperspectral imaging (MacCormac et al.; Caredda et al., 2023), fluorescence imaging and spectrocopy (MacCormac et al.; Valdes et al., 2011; Valdés et al., 2012; Alston et al., 2019), polarization imaging (Liu et al.), optical coherence tomography (Müller et al., 2024), and Raman spectroscopy (Ember et al., 2024).

The advancements in intraoperative optical technologies and their impact on neurosurgical guidance and tissue assessment are presented using what is reported in through three original articles and a review article.

One article focuses on the application of polarization imaging technique (PIT) to separate the microstructure of glioma from healthy tissues (Liu et al.). The authors propose a PIT enhancement method based on a backward scattering 3 x 3 Mueller matrix polarization imaging experimental setup and evaluate its applicability to *ex-vivo* unstained glioma and non-glioma samples. They show that the enhancement effect is practically effective and useful when applied to the images of Mueller matrix elements, especially off-diagonal elements. Two indexes related to the contrast and the detailed texture showed significant improvement in image quality. This PIT image enhancement method was able to greatly improve the contrast, and through a detailed texture information of Mueller matrix images, useful clinical information could be obtained.

The second article investigates the application of intra-operative hyperspectral imaging as a label-free tissue differentiation method (MacCormac et al.). A lightfield hyperspectral camera (Cubert) was integrated into the neurosurgical workflow to allow the surgeon to capture *in-vivo* hyperspectral data (155 bands, 350–1,000 nm) at 1.5 Hz. The system was evaluated in a pre-clinical setup (IDEAL 0) and during brain tumor surgery in one patient (IDEAL 1). Hyperspectral information was acquired from the cerebellum and associated meningioma with minimal disruption to the neurosurgical workflow, showing different spectral fingerprints related to the pathological status. This study opens the doors for further development of hyperspectral imaging that can provide real-time, wide-field, and label-free intra-operative imaging and tissue differentiation.

The third article is related to the guidance for glioma surgery through 5-aminolevulinic acid (5-ALA)-induced fluorescence, and particularly the blue-shifted spectral shape of protoporphyrin IX (PpIX) in relation to the emission peak at 620 nm (Suero Molina et al.). The authors reviewed more than 200,000 spectral images from various tumors measured in almost 600 biopsies of 130 patients and carefully considered the impact of autofluorescence crosstalk (flavin, lipofuscin, NADH and porphyrins derivatives) on PpIX620. This work highlights the complex interaction of various fluorophores in glioma with close emission spectra. But this method may produces an overestimation of PpIX620. There is a need for further investigations to gain a more comprehensive understanding of the spectral complexity in gliomas.

The last article reviews of the use of 5-ALA induced PpIX fluorescence spectroscopy in neurosurgery (Gautheron et al.). It gives an overview of the physics underlying fluorescence in biological tissues and focuses on 5-ALA induced PpIX fluorescence spectroscopy methods (intensity, spectral shape, time-resolved) and describes their specific features (hardware requirements, main processing methods) as well as their strengths and limitations. Finally, it addresses current clinical applications and future directions of 5-ALA induced PpIX fluorescence spectroscopy in neurosurgery.

Overall, the articles reviewed here emphasize that optical technologies provide intraoperative access to various imaging biomarkers that are crucial for clinical patient management during neurosurgery. Optical imaging has the potential to impact surgical guidance technologies.
